# Sex and grooming as exchange commodities in female bonobos’ daily biological market

**DOI:** 10.1038/s41598-021-98894-w

**Published:** 2021-09-29

**Authors:** Simone Anzà, Elisa Demuru, Elisabetta Palagi

**Affiliations:** 1 Behavioral Ecology Department, University of Goettingen, Goettingen, Germany; 2 Laboratoire Dynamique Du Langage, CNRS UMR 5596, University of Lyon 2, Lyon, France; 3 Equipe de Neuro-Ethologie Sensorielle, ENES/CRNL, CNRS UMR 5292, Inserm UMR S1028, University of Lyon/Saint-Etienne, Saint-Etienne, France; 4 Unit of Ethology, Department of Biology, University of Pisa, Pisa, Italy; 5 Natural History Museum, University of Pisa, Pisa, Italy

**Keywords:** Zoology, Animal behaviour

## Abstract

The Biological Market Theory (BMT) posits that cooperation between non-human animals can be seen as a mutually beneficial exchange of commodities similarly to what observed in human economic markets. Positive social interactions are commodities in non-human animals, and mutual exchanges fulfilling the criteria of the BMT have been shown in several species. However, the study of biological markets suffers from methodological limitations that are mainly linked to the difficulty of clearly identifying the currencies and their exchanges in the short-term. Here, we test whether bonobo females are more attractive during their maximum swelling phase, whether they exchange grooming and Genito-Genital Rubbing (GGR) on a daily level of analysis, and whether these daily exchanges fulfil the BMT criteria. Females engaged more in GGR when their sexual swelling was in the maximum phase. Moreover, they exchanged grooming and sex according to the daily “market fluctuations” associated with swelling status. Females in the minimum phase (low-value) increased their probability to engage in GGR with females in the maximum phase (high-value) by grooming them preferentially. In line with the supply/demand law, the female grooming strategy varied depending on the daily number of swollen females present: the higher the number of swollen females, the lower the individual grooming preference. As a whole, our study confirms BMT as a valid model to explain daily commodity exchanges as a function of the temporary value of traders, and underlines the importance of a day-by-day approach to unveil the presence of a biological market when the value of traders frequently changes.

## Introduction

The Biological Market Theory (BMT) posits that cooperation between non-human animals can be seen as a mutually beneficial exchange of commodities (i.e. goods or services) similarly to what is observed in human economic markets^1^. Within this context, a commodity must be monopolizable and cannot be obtained by force, so that individuals should develop an alternative strategy to conflict to get the commodity^2^. Within the same class of traders, individuals are chosen as partners depending on the commodities available on the market, therefore the best strategy is either to increase the value of the commodity offered, or to demand less in return in order to outcompete the other traders^3^. As it occurs in human markets^4^, the exchange rate of commodities in a Biological Market depends on the law of supply and demand: when a certain commodity is rare, competition over it is high, as its value is perceived as being high as well. Under these circumstances, the other individuals are inclined to “pay more” to get the rare high-value commodity^3^.

Social interactions are a valuable commodity in non-human animals, and mutual exchanges fulfilling the criteria of the BMT have been shown in several species (cleaners-client fishes^5^; red bishops, *Euplectes orix*^6^; wood mice, *Apodemus sylvaticus*^7^; meerkats, *Suricata suricatta*^8^).

The BMT has also been used to explain several types of short- and long-term exchanges in non-human primates (hereafter primates), with most studies focusing on allo-grooming as a commodity to exchange (e.g., for sex in sifaka, *Propithecus verreauxi*^9^; for sex in gibbons, *Hylobates lar*^10^; for sex in chimpanzees, *Pan troglodytes*^11^; for grooming in bonobos, *Pan paniscus*^12^). Allo-grooming is a frequent and valuable behaviour among primates where it can serve the functions of reinforcing social bonds^13,14^, fostering coalitions^15^, and reducing tension^16^. As highlighted by Sánchez-Amaro and Amici^17^, the study of biological markets in primates suffers from theoretical and methodological limitations that are mainly linked to the difficulty of identifying clear exchanges in cognitively and socially complex species. Indeed, primates are capable of keeping track of both short- and long-time exchanges^18^ and live in markets where social commodities are not only highly variable but also strongly interconnected^19^. Concerning the temporal scale, there is a total lack of studies focussing on the exchanges on a daily level of analysis.

Here, we focus on the bonobo (*Pan paniscus*) to fill this gap by exploring the possible existence of a biological market regulating the exchanges of social interactions in females living in two stable social groups. To reach this goal, we focussed our analyses on the daily fluctuation of grooming and socio-sexual interactions as a function of the swelling status of actor and receiver. As far as we know, it has never been demonstrated that the Biological Market Theory explains the exchange of commodities in bonobos, although it has been hypothesized^20^.

Together with chimpanzees (*Pan troglodytes*), bonobos are human closest living relatives. The two *Pan* species live in multi-male/multi-female social groups characterized by fission–fusion dynamics and show male philopatry and female dispersal^21^. This dispersion pattern causes a higher level of relatedness among males than among females, nevertheless bonobo females are highly cohesive and share strong affiliative bonds^22–24^. Female cohesiveness and affiliation allow bonobo females to be dominant over males—or co-dominant according to some authors^25^—which is a notable exception in a male-philopatric species^26^. Female bonds are established and maintained through a wide array of affiliative behaviour such as play^27^, consolation^28^, agonistic support^23^, allo-grooming^26^, face engagement^29^, and socio-sexuality^30^. Bonobos are well known for making use of sexual behaviour for social purposes and, particularly, to decrease social tension during competitive contexts, such as food distribution or aggressive interactions^31^. Bonobo females frequently engage in Genito-Genital Rubbing (GGR), a socio-sexual behaviour during which females embrace each-other, frequently face to face, and rub their genitals by moving their hips side to side^32^. Recently, Moscovice et al.^30^ showed that GGR plays an essential role in enhancing female cooperation as this behaviour stimulates the release of oxytocin. Moreover, this face-to-face interaction favours the perception and replication of facial expressions through the phenomenon of Rapid Facial Mimicry (RFM^33^). RFM is an unconscious phenomenon that represents a basic layer of empathy^34^ and is a powerful mechanism that helps establishing an emotional bridge between two subjects.

Socio-sexual interactions involving females are enhanced by the presence of the maximum sexual swelling, a conspicuous and temporary signal that in bonobos is highly attractive for both males and females^35,36^. The ano-genital region of female bonobos goes through changes in size and turgidity along the menstrual cycle, and the attractiveness changes consequently, reaching its peak during the maximum swelling phase^35,36^. Compared to other species, bonobos’ maximum sexual swelling is not a reliable signal of ovulation^37^ and can even be present during anovulatory periods, such as pregnancy and lactation^26^. It has been proposed that bonobos’ maximum swelling features—together with a posture possibly functioning as amplifier signal^38^—might have been selected to increase female-female sexual interactions^35^. Through socio-sexuality, bonobo females get integrated in a group, establish affiliative bonds and increase social cohesiveness which, in turn, allow them to form coalitions that increase their social status^26,27^.

In the present study, we aimed at testing whether daily grooming and GGR rates across adult females are affected by the different value that females acquire based on their swelling phase, and whether such commodities are exchanged according to the Biological Market Theory (BMT).

### Prediction 1—basic assumption

For a biological market to be established, it is necessary to first demonstrate that a desirable resource is present. If the maximum swelling phase increases the attractiveness of females and the motivation to engage in GGR^35,36^, we predict GGR rates to be higher when at least one of the females in the dyad is in the maximum swelling phase.

### Prediction 2

If grooming is a commodity that is exchanged on a daily basis, we predict that grooming rates show daily fluctuations following the supply/demand law. Specifically, we expect female grooming rates to decrease as the number of available females in maximum swelling phase increases (*Prediction 2a*). Moreover, we expect dyadic grooming preference to vary on a daily basis as a function of actor’ and receiver’s swelling state (*Prediction 2b*).

### Prediction 3

If grooming and GGR are commodities that are exchanged according to the BMT, we expect the daily frequency of GGR to fluctuate as a function of the number of females showing maximum swelling (supply/demand law) (*Prediction 3a*). Moreover, we expect GGR to be influenced by both the daily grooming rate (commodity) and the swelling phase (temporary value). Specifically, we predict the positive effect of grooming on the frequency of GGR to be maximum when the actor is in the minimum and the receiver is in the maximum swelling phase (*Prediction 3b*).

## Methods

### Ethic statement

This study was approved by Universities of Pisa and Parma (Animal Care and Use Board). Since the study was purely observational with no human-animal interaction and/or manipulation whatsoever, the committee waived the need for an ethical permit.

### Study sites and subjects

The study was part of a long-term research project on bonobo social behavior. Data were collected at the Apenheul Primate Park (Apeldoorn, The Netherlands) and at La Vallée des Singes (Romagne, France) where bonobos lived in inter-connected indoor enclosures with free access to an outdoor naturalistic forested island (Supplementary Table S1, Supplementary Information: Housing and living condition). In Apenheul, observations were carried out between August and November 2009 by two observers (one being ED) for 6 days a week, seven hours per day. The group was composed by 5 adult females, 2 adult males, and 3 immature subjects (see Supplementary Information).

In La Vallée des Singes, observations were carried out between June and August 2012 by two observers (one being ED), 6 days a week from 9:00 to 18:00. The group was composed by 5 adult females, 4 adult male, and 1 immature subject (see Supplementary Information).

### Data collection

In Apenheul, behavioural data were collected by using a voice-recorder and were then transcribed on database sheets. The techniques used were Focal Animal Sampling (30-min/Focal for a total of 25 h per individual) and All Occurrences Sampling (a total of 502 h)^39^.

In La Vallée des Singes, behavioural data were collected by using camcoders and by applying the Focal Group Sampling method^39^ for a total of 166 h of videos. The Focal Group Sampling method consists in randomly selecting a sub-group of subjects and recording their behaviours. In our case, we applied this methodology to collect the videos. Videos were then analysed and transcribed on database sheets. The techniques used to analyse the videos were: Focal Animal Sampling (20 h/individual) and All Occurrences Sampling (200 h)^39^.

For both groups, allo-grooming sessions were collected through the Focal Animal Sampling method and were used to establish grooming preference. Sexual and agonistic interactions were collected through the All-Occurrences Sampling method and the latter were used to determine group hierarchy (see below for hierarchy estimation). Kinship through the maternal line was known.

Data were collected and analysed according to a blind coding protocol in which the observers were not aware of the hypotheses and predictions that would have been tested. Before systematic data collection and video analyses, the observers and coders underwent a training phase during which they followed the same focal animal simultaneously and then compared data. The training phase lasted until the Cohen’s Kappa values measured for all the behaviours defined in Supplementary Table 2 reached 0.85^40^.

### Definition of the sexual swelling phases

Daily observations of perineal swelling and menstruation were carried out by the bonobo keepers. Keepers from Apenheul Primate Park recorded daily appearance of sexual swelling stage, according to the following rating system: 1 (maximum detumescence for noncycling females); 2 (minimum swelling phase for cycling females); 3 and 4 (intermediate swelling phase for cycling females); and 5 (maximum swelling phase for cycling females). Using the same parameters, keepers from La Vallée des Singes recorded daily appearance of sexual swellings stage according to the following rating system: 1 (maximum detumescence for noncycling females); 2 (minimum swelling phase for cycling females); 3 (intermediate swelling phase for cycling females); and 4 (maximum swelling phase for cycling females). For data unification, we standardized sexual swelling phases in two categories: minimum (phase 1 + 2) and maximum swelling phase (Apenheul: phase 5; La Vallée des Singes: phase 4). For each adult female showing monthly variations in the swelling cycle (Apenheul group: N = 3; La Vallée group: N = 4—See Supplementary Table 1), the sexual swelling status was recorded by the zoo keepers every day, therefore we analyzed the dyadic grooming preference on a daily basis.

### Calculation of daily grooming preference

The daily grooming preference was calculated by the daily grooming duration (A grooms B) divided by the sum of the daily directional grooming duration performed by the same individual towards all other subjects including B [A grooms (B + C + D)]. The daily grooming preference therefore expresses the proportion of time that one individual spends grooming another one, weighted on the total amount of grooming provided by the same subject on a daily basis. Our study focused on individuals offering grooming, so polyadic grooming sessions were split into dyadic grooming sessions when the focal individual was receiving grooming from multiple partners. It is nevertheless worth noting that polyadic grooming sessions are relatively infrequent in bonobos and very rare among bonobo females^41^. Moreover, there is a positive correlation between the dyads involved in dyadic and in polyadic session^41^.

### Hierarchy

We evaluated hierarchical relationships on the basis of dyadic and decided conflicts that occurred over the whole period of observation by using a winner/loser matrix to assess the rank according to the Normalized David’s Scores (NDS^42^). NDS were calculated on the basis of a dyadic dominance index (Dij) in which the observed proportion of wins (Pij) is corrected for the chance occurrence of the observed outcome. The chance occurrence of the observed outcome was calculated on the basis of a binomial distribution with each animal having an equal chance of winning or losing in every agonistic encounter^42^. The correction is necessary when the interaction numbers greatly differed across dyads.

### Statistics

To compare GGR frequencies of dyads involving females in the minimum swelling phase to dyads involving at least one female in the maximum swelling phase, we ran a paired randomization test at the dyadic level with a number of 10,000 shuffles (allowing us to achieve an accuracy of 0.001 of the probability values) to avoid errors due to non-independence of the data^43^. The test allowed us to understand whether the maximum swelling condition is attractive for other females (*Prediction 1—Basic assumption*). We estimated the individual frequency of GGR per day for each swelling phase (e.g., number of GGR_MaxSwelling_/number of days in maximum swelling). We used the software Resampling Procedures 1.3 by David C. Howell (freeware), that provides a t-value (difference between the means of the samples, standardized by the standard error) when comparing two dependent groups, with the probability to obtain such values under the null hypothesis.

We ran all Generalized Linear Mixed Models (GLMM) models in R (version 4.0.1^44^) using the RStudio interface (version 1.3.959^45^). Prior to fitting the model, all the covariates were z-transformed to a mean of 0 and a standard deviation of 1 to achieve more easily interpretable estimates and facilitate model convergence^46^. To rule out collinearity we determined Variance Inflation Factors (VIFs) using the function *vif* of the R package *car* (version 3.0-3^47^) applied to a standard linear model excluding the random effects and the interactions. Both Model 1 and Model 2 presented below have been tested with the inclusion of female parity as factor shaping grooming preference and GGR. However, due to very high collinearity with actor and receiver’s age (VIF *parity.act* = 64.18; VIF *parity.rec* = 76.84) we excluded them from all the analyses. After removal of parity, all the VIF values were considered acceptable (maximum VIFs across models = 3.31). Model stability was assessed using a function that excluded the levels of the random effects one at a time^48^. For all models, we performed a full vs control model comparison, with the control model containing only random effects, random slopes and control predictors to test whether the inclusion of explanatory predictors improved model fit. Tests of the individual predictors were derived using likelihood ratio tests^49^ (R function *drop1* with argument ‘test’ set to “Chisq”). We conducted post-hoc comparisons using Tukey tests, and report only significant tests.

We ran Model 1 to test *Prediction 2a* (effect of the daily number of available females showing maximum swelling on grooming preference) and *Prediction 2b* (effect of actor’ and receiver’s swelling state on daily dyadic grooming preference). Model 1 investigated whether daily grooming preferences (as dependent variable) varied depending on the following predictors: the daily number of females in the maximum swelling condition, the sexual swelling condition (binomial) of actor and receiver as well as their two-way interaction, and the group (binomial). *Parity* was not included due to high collinearity with *age* (see above). Considering the response range 0–1, we modelled it with a Beta error distribution and logit link function after data transformation which excluded exact values of 0 and 1^50^. The number of females with the maximum sexual swelling included all the studied females that a given actor could access a given day. We included as control predictors the two-way interaction of actor and receiver’s age (numeric) and the two-way interaction of actor and receiver’s rank (continuous variable). We included the two-way interaction of actor and receiver’s rank to control for a possible influence of rank similarity/dissimilarity on the daily grooming preference. We included the two-way interaction of actor and receiver’s age to control for a possible influence of age similarity/dissimilarity. We removed non-significant two-way interactions and tested their main predictors. Identity of actor, receiver and dyad were included as random effects. We excluded random slopes due to convergence issues. Model 1 included 94 cases of daily grooming preferences involving 16 different dyads. In a given dyad AB, the grooming preference of subject A towards B is not equal to the preference of B towards A, allowing our model to estimate the effect of AB controlling for BA, and vice versa.

We ran Model 2 to test *Prediction 3a* (effect of the daily number of available females showing maximum swelling on daily GGR frequency) and *Prediction 3b* (effect of daily grooming preference on the daily frequency of GGR depending on actor’ and receiver’s swelling phase). Model 2 investigated the effect of the number of swollen females, daily grooming preference (continuous variable) and sexual swelling (binomial) on the daily occurrence of GGR (numeric), by considering the directionality of the interaction. We modelled the response with a Gamma error distribution and log link function. To calculate the occurrence of GGR, we analyzed the successful sexual invitations (i.e., those leading to a GGR) performed by the actor towards the receiver. Gestures, body postures and facial expressions used as sexual invitations are listed and described in Supplementary Table S2. All the GGR events not preceded by any clear sexual invitation were halved and assigned to both the actor and the receiver. We included as test predictors the following variables: the daily number of females in the maximum swelling condition, the three-way interaction of daily grooming preference and sexual swelling condition of both the actor and receiver, and the group. *Parity* was not included due to high collinearity with *age* (see above). We included as control predictors the two-way interaction of actor and receiver’s age and the two-way interaction of actor and receiver’s rank. We removed the not significant two-way interaction *age*_act_*age_*rec*_ and repeated the model to assess the effect of main age predictors. Identity of actor, receiver and dyad were included as random effects and the *number of swollen females* and *grooming preference* as random slopes. Because Model 2 did not converge, we excluded from the random slopes *grooming preference* and re-ran Model 2 successfully. Model 2 included 74 cases involving 13 dyads.

## Results

### Genito-genital rubbing

Females engaged significantly less in GGR when they were both in the minimum swelling phase (mean ± SD: 0.32 ± 0.60), compared to when at least one female of the dyad was in the maximum swelling phase (mean ± SD: 0.61 ± 0.69) (Randomization paired t_9_ = − 2.275, *P* = 0.027) (*Prediction 1 supported*).

### Model 1—﻿daily grooming preference

**Figure 1:**
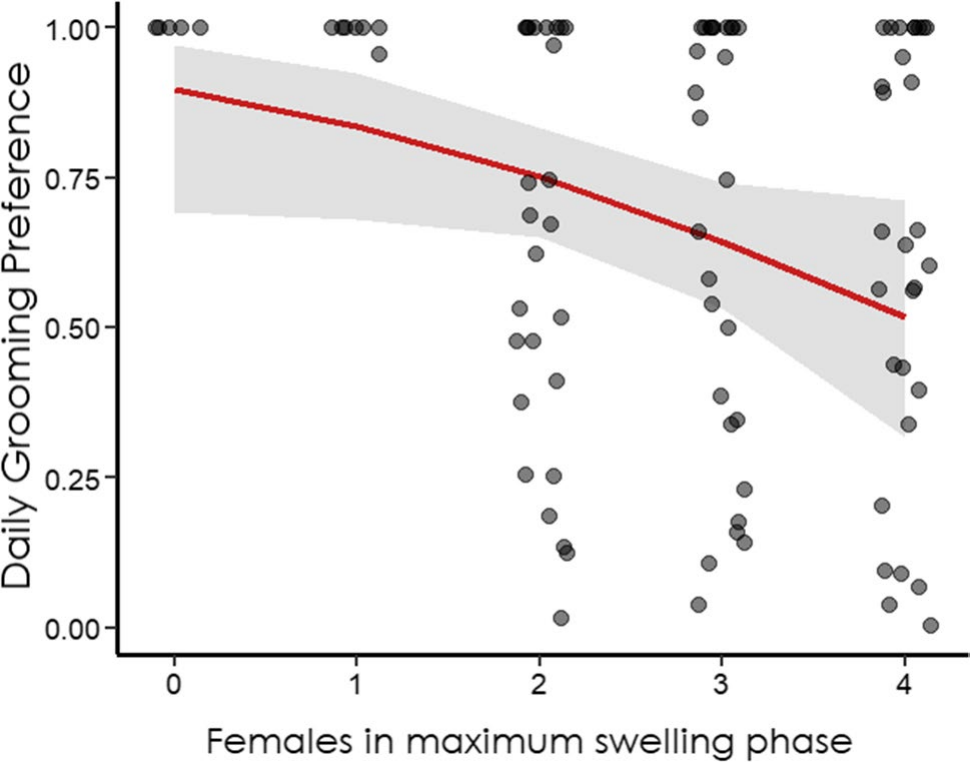
Effect of the number of females in maximum swelling phase on daily grooming preference (red solid line) at the average of all other predictors. Gray band indicates 95% Confidence Intervals. Data points are plotted using *jitter* function at 0.15 (width) to avoid complete overlapping and improve graph interpretability.

**Figure 2:**
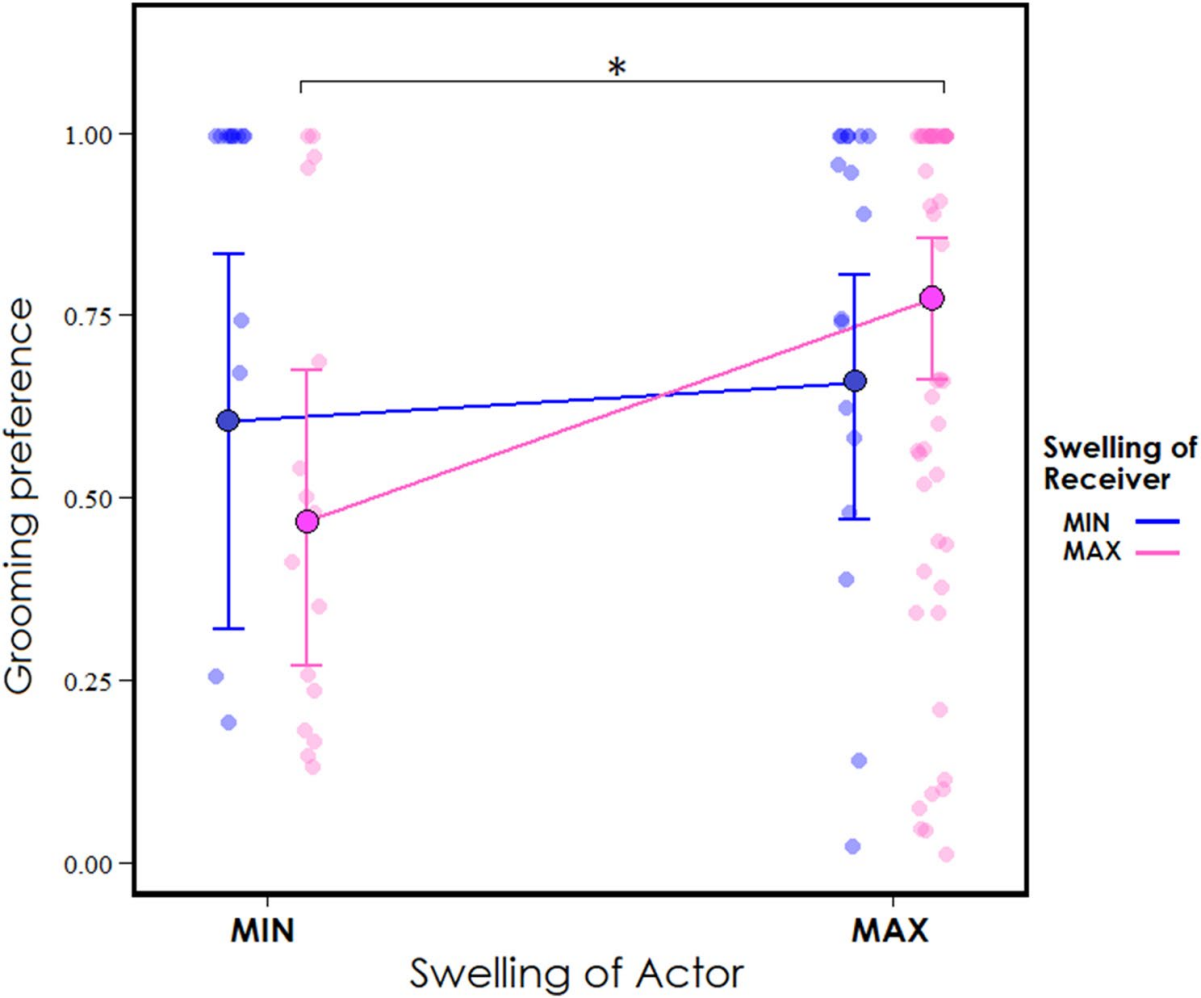
Predicted effect of the interaction of actor and receiver’s swelling state on the dyadic grooming preference. Dots with borders and whiskers indicate estimated mean and standard errors of categorical responses, while colored lines indicate variation in receiver’s effect according to actor’s swelling. The figure is plotted with all other predictors at their average value. Dots without borders indicate raw data plotted with *jitter* function at 0.1 (width). Parentheses indicate significant post-hoc comparison.

Overall, the predictors *number of swollen females,* the two-way interaction *swelling*_act_*swelling_*rec*_*,* and *group*, had an effect on the daily grooming preference, after controlling for rank and age effect (full vs control model comparison Model 1: *χ*^2^ = 11.92, df = 5, *p* = 0.035, pseudo-R^2^ = 0.32). The model did not include the non-significant interactions of *age*_act_*age_*rec*_ (*p* = 0.746) and *rank*_*act*_ *rank_*rec*_ (*p* = 0.726) and assesses the effect of age and rank as main control predictors. The daily grooming preference was significantly affected by the number of swollen females available the same day: the higher the number of swollen females, the lower the daily grooming preference (*Prediction 2a supported*) (Table 1, Fig. 1). An increase of 1 in the number of females in the maximum swelling phase decreased the probability of grooming from 88 to 80%. Moreover, the two-way interaction *swelling*_act_*swelling_*rec*_ had a significant effect on the daily grooming preference and increased the likelihood of grooming of 2.27 times (+ 69%, Table 1). After removal of non-significant interactions, all main control predictors were not significant. We ran post-hoc comparisons to estimate the differences in the effect of actor’ and receiver’s swelling condition on daily grooming preferences (Fig. 2). Specifically, when both the actor and the receiver were in the maximum swelling condition the dyadic grooming preference was higher compared to when the actor was in the minimum and the receiver was in the maximum swelling condition (*p* = 0.009 Tukey adjusted, Fig. 2) (*Prediction 2b supported*).

**Table 1:** Variables in Model 1 explaining the daily grooming preference while controlling for rank and age of both actor and receiver. LTR = likelihood-ratio test.

Fixed variables	Estimates	SE	Z-value	2.5% C.I	97.5% C.I	LRT	df	*P*
Intercept	1.95	0.50	3.93	0.98	2.92	–	–	–
**N. of swollen females**	**−** **0.52**	**0.26**	**−** **2.03**	**−** **1.02**	**−** **0.02**	**4.41**	**1**	0.036 *
^a^**Swelling act**_**(max)**_ * Swelling rec_**(max)**_	**1.13**	**0.57**	**1.98**	**0.01**	**2.25**	**3.94**	**3**	0.047 *
^a^Swelling act_(max)_	0.22	0.52	0.43	− 0.80	1.24	–	–	–
^a^Swelling rec_(max)_	− 0.56	0.60	− 0.94	− 1.74	0.62	–	–	–
^b^Group2	− 0.13	0.51	− 0.25	− 1.13	0.88	0.06	1	0.805
^c^*Rank act*	0.06	0.22	0.29	− 0.36	0.49	0.08	1	0.773
^c^*Rank rec*	0.47	0.28	1.69	− 0.07	1.02	3.11	1	0.078
^c^*Age act*	− 0.11	0.22	− 0.51	− 0.54	0.31	0.24	1	0.622
^c^*Age rec*	− 0.23	0.24	− 0.97	− 0.70	0.23	0.98	1	0.322

Control variables are in italics and explanatory variables with significant *P* values are in bold and marked with *

^a^Swelling condition “min” as reference category.

^b^Group1 as reference category.

^c^z-transformed to a mean of 0 and a standard deviation of 1.

### Model 2 – Sex, swelling and grooming

**Figure 3:**
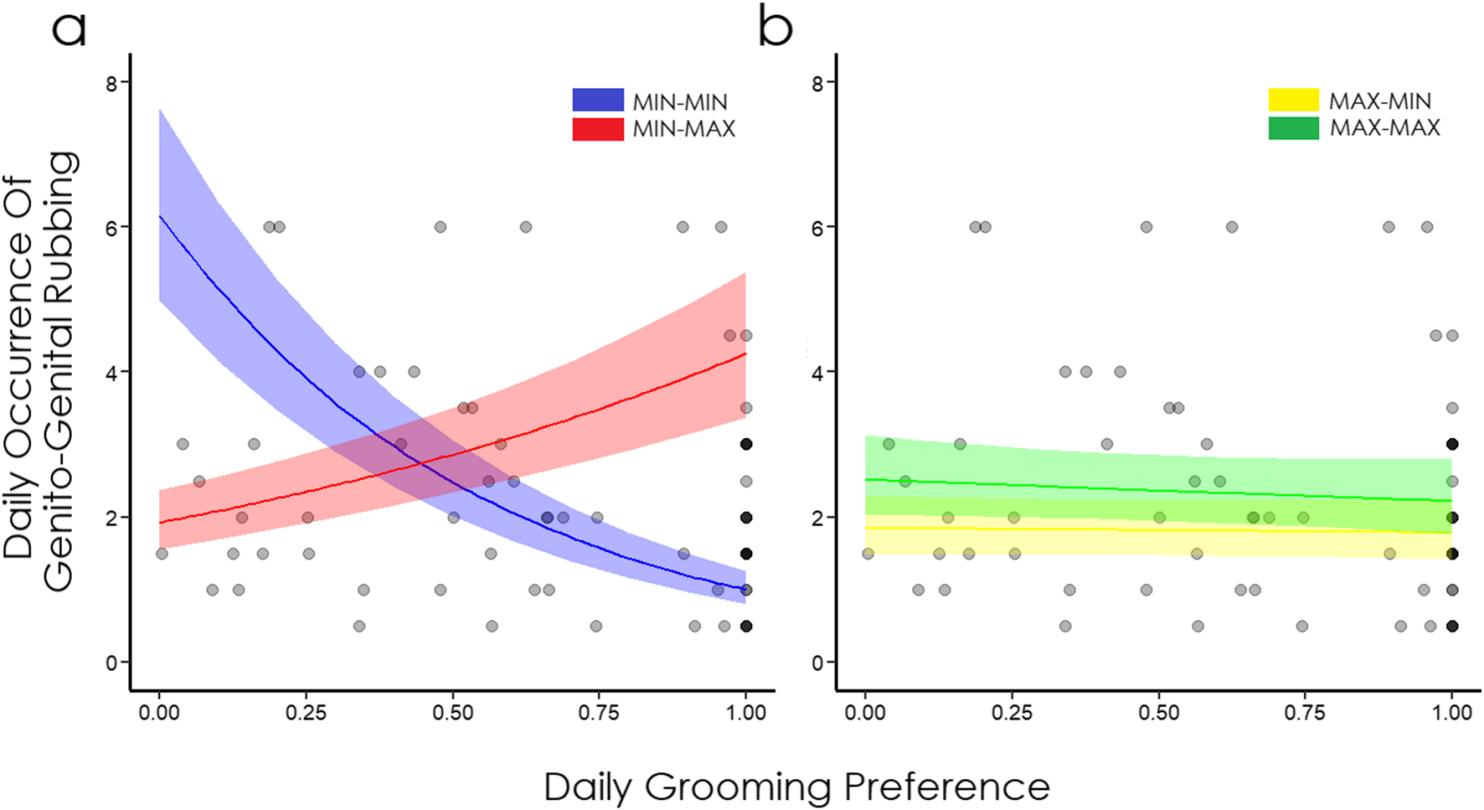
Effect of the three-way interaction grooming*swelling_act_*swelling_*rec*_. (**a**) Indicates actor in the minimum swelling phase when interacting with a receiver in the minimum swelling phase (in blue) or with a receiver in the maximum swelling phase (in red). (**b**) Indicates actor in the maximum swelling phase when interacting with a receiver in the minimum swelling phase (in yellow) or with a receiver in the maximum swelling phase (in green). Shaded bands indicate 95% Confidence Intervals. Darker points represent overlapping of several data points.

Overall, the number of swollen females and the three-way interaction Grooming*Swelling_act_*Swelling_*rec*_ had an effect on daily GGR distribution after controlling for rank and age (full vs control model comparison of Model 2: *χ*^2^ = 22.75, df = 10, *p* = 0.011, conditional R^2^ = 0.40). The model did not include the non-significant interaction of *age*_*act*_ *age_*rec*_ (*p* = 0.707) and assesses the effect of age as main predictors. The daily GGR occurrence was significantly affected by the number of females in the maximum swelling phase: the higher the number of swollen females, the lower the GGR occurrence (− 29.83% probability of GGR with an increase of 1 female in maximum swelling phase; *p* = 0.002, Table 2) (*Prediction 3a supported*). Moreover, the daily GGR occurrence was significantly affected by the three-way interaction of daily grooming preference and sexual swelling phase of both the actor and the receiver (*p* = 0.011; Table 2, Fig. 3). Actors in maximum swelling phase (MAX) had 1.02 times higher (+ 2.94%) odds of engage in GGR than actors in minimum swelling phase (MIN), while receivers in MAX had 1.90 times higher (+ 90.33%) odds than receiver in MIN. Additionally, receivers in MAX had 1.84 times higher (+ 84.89%) odds of engage in GGR when compared with actors in the same condition, highlighting the role of receivers in accepting sexual contacts. Interestingly, an increase of 1 SD of grooming preference when actor and receiver where both in the minimum swelling phase (MIN-MIN) was associated with an effect size of 0.54 (− 45.70%), suggesting that grooming is not exchanged with GGR when both females are in the minimum swelling phase. Consistently with the Biological Market Theory, the grooming preference of actor in minimum and receiver in maximum swelling phase (MIN–MAX) had an effect 1.32 times higher (+ 32.01%) when compared with MAX–MIN, and 4.43 times higher (+ 342.61%) when compared with MIN-MIN.

**Table 2:** Variables in Model 2 explaining the occurrence of daily GGR while controlling for rank and age of both actor and receiver.

Fixed variables	Estimates	SE	Z-value	2.5% C.I	97.5% C.I	LRT	df	*P*
Intercept	1.51	0.07	21.38	1.37	1.65	–	–	–
**N. of swollen females**	**−** **0.35**	**0.04**	**−** **9.22**	**−** **0.43**	**−** **0.28**	**9.20**	**1**	**0.002**
^a^^,b^Grooming * Swelling_**act(max)**_ * Swelling_**rec(max)**_	**−** **0.91**	**0.07**	**−** **13.78**	**−** **1.04**	**−** **0.78**	**6.41**	**1**	**0.011**
^a^^,b^Grooming * Swelling_act(max)_	0.60	0.06	9.36	0.47	0.72	–	–	–
^a^^,b^Grooming * Swelling_rec(max)_	0.88	0.06	13.94	0.75	1.00	–	–	–
^a^^,b^Swelling_act(max)_ * Swelling_rec(max)_	− 0.40	0.07	− 5.77	− 0.53	− 0.26	–	–	–
^b^Grooming	− 0.61	0.06	− 10.48	− 0.72	− 0.50	–	–	–
^a^Swelling_act(max)_	0.03	0.07	0.43	− 0.10	0.16	–	–	–
^a^Swelling_rec(max)_	0.64	0.07	9.34	0.51	0.78	–	–	–
^b^Group2	0.20	0.07	2.79	0.06	0.34	0.33	1	0.563
^c^Rank act * rank rec	0.37	0.05	6.84	0.26	0.47	**7.03**	**1**	**0.008**
^c^*Rank act*	− 0.37	0.05	− 6.99	− 0.47	− 0.26	–	–	–
^c^*Rank rec*	− 0.51	0.06	− 9.00	− 0.62	− 0.40	–	–	–
^c^***Age act***	**0.48**	**0.05**	**8.88**	**0.38**	**0.59**	**10.77**	**1**	**0.001**
^c^*Age rec*	0.21	0.05	3.94	0.11	0.32	2.15	1	0.142

*LTR* likelihood-ratio test.

Control variables are in italics, variables with significant *P* values are in bold and marked with *. Grooming = daily dyadic grooming preference. Swelling_act,rec_ = sexual swelling condition of actor or receiver.

^a^Dummy coded with “min” swelling condition as reference category.

^b^Dummy coded with Group1 as reference category.

^c^z-transformed to a mean of 0 and a standard deviation of 1.

Post-hoc analyses confirmed the differences in the effect of grooming preference on daily GGR occurrence according to different sexual swelling conditions. Specifically, when the actor was in the minimum swelling condition and the receiver was in the maximum swelling condition, grooming preference increased daily GGR occurrence when compared with both actor and receiver in the minimum swelling condition (MIN–MAX vs MIN-MIN: p < 0.001, Fig. 3). After removing the non-significant two-way interaction *age*_act_*age_*rec*_, only the main control predictor *age*_*act*_ was significant (*p* = 0.001, Table 2, Supplementary Figure 1). Moreover, the two-way interaction of the control predictors *rank*_act_*rank_*rec*_ was significant (*p* = 0.008, Table 2, Supplementary Figure S2), with a positive effect on the daily occurrence of GGR.

## Discussion

Taken together, our results confirm the key role of sexual swelling in bonobos and provide new insights on the exchange of grooming with sex on a daily basis. More particularly, our study shows that grooming and sex represent valuable exchange commodities for bonobo females and that their exchange fluctuates depending on females’ sexual swelling status. These daily fluctuations can be explained by the supply/demand law^1^. In line with other studies on sexual swelling and socio-sexual behaviour in bonobos^35,36^, our results confirmed that females engaged significantly more in Genito-Genital Rubbing (GGR) when at least one female of the dyad was in the maximum swelling phase, compared to when both of them were in the minimum swelling phase (*Prediction 1*). When in the maximum swelling phase, females are more attractive for both males and females and tend to be invited more frequently to engage in sexual interactions than females in the minimum swelling phase^26^. This is in line with the hypothesis proposing that the peculiar features of bonobo females’ sexual swelling, and in particular its extremely long duration and presence during anovulatory periods, might have been selected to increase female-female sexual interactions^35^. Socio-sexuality in bonobo females help establishing and maintaining strong social bonds which, in turn, allow them to form coalitions that increase their social status and centrality^30^.

According to the Biological Market Theory derived from the Market Theory applied in economics, the exchange rate of commodities depends on the supply/demand law, and when a certain commodity becomes common, its perceived value decreases. Consistent with the supply/demand law, the daily grooming preference was significantly affected by the number of females showing the sexual swelling at the maximum phase during the same given day: the higher the number of swollen females, the lower the daily grooming preference (*Prediction 2a,* Fig. 1). Interestingly, when both the actor and the receiver were in the minimum swelling phase, and there were no females available in the maximum swelling phase, the grooming preference was very high. Via Model 1 we considered the directional grooming preference and we included the preference of both dyads AB and BA. Therefore, our results suggest that females exchanged grooming for grooming when both in the minimum swelling phase. Moreover, the significant effect of the interaction *swelling*_act_*swelling_*rec*_ showed higher grooming preference when both the actor and the receiver were in the maximum swelling phase, compared to when the actor was in the minimum and the receiver was in the maximum swelling phase. These results confirm the higher attractiveness of females showing the maximum swelling phase (*Prediction 1, 2b*), suggest that females tend to exchange grooming for grooming when their value is “low” (MIN) and that high-value females (MAX) prefer “high-value” grooming partners as well.

Our results on the effect of the number of swollen females on grooming preference are in line with a study on male–female grooming in captive chimpanzees^11^ showing that females in the maximum swelling phase receive more grooming. Although our investigation focussed on female-female sexual interactions, our results are in line with those of chimpanzees given that the highest daily preference was achieved when both actor and receiver were in the maximum swelling phase. The results coming from the two *Pan* species are comparable considering the high attractiveness of the maximum sexual swelling in both chimpanzees and bonobos although in chimpanzees the sexual swelling is attractive for males only, while in bonobos this signal is attractive for both sexes.

In many primate groups, higher-ranking subjects typically receive more grooming than lower-ranking ones^51–53^ and most of the grooming occurs between subjects with similar rank^54^. Among bonobos, the distribution of grooming is related to several variables (e.g., age, rank, and sex) and there are some indications that high-ranking and older females are preferred grooming partners^55^. Subjects within the same group may therefore compete for grooming access to high-ranking groupmates, since those can be the best coalition partners^52,54^. Alternatively, a grooming distribution related to rank can be explained by females’ preference in grooming subjects of similar social class (e.g., rank and age) as an adaptive strategy derived from greater compatibility between subjects^56^. These mechanisms are beyond the aim of our investigation but can still drive the variation of grooming preferences and mask the effect of the daily market. For this reason, we included in our models rank and age of both actor and receiver as control predictors. However, our results on the daily grooming preference showed no effect of actor and receiver’s rank and age, suggesting that the factors shaping grooming preference among bonobo females on a daily basis may not coincide with those affecting such preference on the long term. Moreover, it has been showed that the steepness of the hierarchy varies between different bonobo groups in captivity^20,25^. Particularly, these studies demonstrated that grooming was preferentially directed towards dominant individuals in those groups where the hierarchy was very steep, whereas grooming was more reciprocal in those groups showing a mild hierarchy. It is worth noting that the steepness of our study groups was extremely low (Apenheul: 0.346; La Vallée: 0.305 unpublished data).

The daily occurrence of GGR was significantly affected by the number of females in the maximum swelling phase: the higher the number of swollen females, the lower the daily occurrence of GGR (*Prediction 3a*). Post-hoc analyses revealed no significant effect of grooming preference on the daily occurrence of GGR when the actor was in the maximum swelling condition and the receiver was in the maximum or minimum swelling condition (Fig. 3b). On the other hand, when an actor in the minimum swelling phase preferentially groomed a receiver in the maximum swelling phase, it significantly increased the probability of engaging in GGR compared to when the receiver was in the minimum swelling phase (*Prediction 3b*, Fig. 3a). This might indicate that: (1) when both partners are in the minimum swelling phase, none of them has the “economic power” to influence the exchange of goods and grooming is therefore not exchanged with sex (Fig. 3a; MIN-MIN), and that (2) lower-value females (MIN) need to provide more grooming to get the access to higher-value partners (Fig. 3a; MIN–MAX). However, this strategy seems to work only when is the receiver (of grooming/sexual invitations) who benefits from the mismatch, especially if we consider that an increase of grooming performed by a high-value actor does not increase the chances of engaging in sexual interactions: a high-value actor has a constant chance of engaging in sexual interactions despite receiver’s value (Fig. 3b; MAX–MIN, MAX-MAX). The results from Model 1 and Model 2 are consistently stressing that grooming can be performed to balance the mismatch in females’ value, and when the partners have equal value, grooming can be exchanged for grooming as well.

Females in the minimum swelling phase exhibit a short-term strategy of exchanging commodities shaped by the supply/demand law and receiver’s value. Given that females in the maximum swelling phase are more sexually (and therefore socially) attractive, females in the minimum swelling phase must augment the commodity they provide to increase their possibility of engaging in sexual interactions with higher-value females. Furthermore, by strategically offering more grooming, lower-value females can even outcompete higher-value ones to gather “sexual favours” from the most attractive partners.

In conclusion, our study confirms the BMT in explaining the daily exchange of commodities and its fluctuations as a function of the temporary value of traders, and underlines the importance of a day-by-day approach to unveil the presence of a biological market when the value of traders rapidly changes.

## Supplementary information

Supplementary Information 1.

Supplementary Information 2.

## Data Availability

The raw data supporting the conclusion of this article are provided as supporting material.
