# Publisher Correction: Sex and grooming as exchange commodities in female bonobos’ daily biological market

**DOI:** 10.1038/s41598-021-00136-6

**Published:** 2021-10-12

**Authors:** Simone Anzà, Elisa Demuru, Elisabetta Palagi

**Affiliations:** 1 Behavioral Ecology Department, University of Goettingen, Goettingen, Germany; 2 Laboratoire Dynamique Du Langage, CNRS UMR 5596, University of Lyon 2, Lyon, France; 3 Equipe de Neuro-Ethologie Sensorielle, ENES/CRNL, CNRS UMR 5292, Inserm UMR S1028, University of Lyon/Saint-Etienne, Saint-Etienne, France; 4 Unit of Ethology, Department of Biology, University of Pisa, Pisa, Italy; 5 Natural History Museum, University of Pisa, Pisa, Italy

Correction to: *Scientific Reports* 10.1038/s41598-021-98894-w, published online 29 September 2021

In the original version of this Article Simone Anzà and Elisa Demuru were omitted as equally contributing authors.

The original Article has been corrected.

