# Supplementary material for: Sex and grooming as exchange commodities in female bonobos’ daily biological market
Source: Sci Rep. 2021 Sep 29;11:19344. doi: 10.1038/s41598-021-98894-w (PMC8481276; doi:10.1038/s41598-021-98894-w)
Supplement: Supplementary file 2 — Supplementary Information 2. [file 41598_2021_98894_MOESM2_ESM.docx]

# **Supplement to:**

**Sex and grooming as exchange commodities in female bonobos’ daily biological market**

**This supplement includes:**

**Housing and living conditions**

**Supplementary Table S1-2** Group Composition and sexual behavior ethogram.

**Supplementary Figure S1-2** Effect of control variables

**Housing and living conditions**

The colony from the Apenheul Primate Park was hosted in a naturalistic island of 5,000 m^2^ connected with multiple indoor enclosures of 230 m^2^. All the enclosures were equipped with environmental enrichments allowing the animals to move freely in all three dimensions. The subjects were fed four times per day (9:00, 12:45, 15:00, 17:30) and most of the food was scattered on the ground. Water was available *ad libitum* and several environmental enrichments were provided also in the outdoor facility. The subjects showed no stereotypic or aberrant behaviour.

Similarly, the colony from La Vallée des Singes was hosted in an outdoor area of about 8,500 m^2^ connected with indoor enclosures of approximately 500 m^2^. All the enclosures were equipped with environmental enrichments allowing the animals to move freely in all three dimensions. To encourage foraging activity, the keepers were spreading food around the external facility before the subjects were allowed to go out, around 9:00. The subjects were fed six times per day (11:15, 12:30, 14:00, 15:00, 16:00, 17:00) and most of the food was scattered on the ground. Water was available *ad libitum* and several environmental enrichments were provided. The subjects showed no stereotypic or aberrant behaviours.

**Supplementary Table S1.** Composition of the bonobo groups hosted in **a)** 2009 at the Apenheul Primate Park (Apeldoorn, The Netherlands) and **b)** in 2012 at the La Vallée des Singes (Romagne, France). (*) indicates the adult females included in the analysis.

| Name | Kinship | Sex | Age class | Year of birth |
| --- | --- | --- | --- | --- |
| Jill* | Lingala’s mother | F | Adult | 1985 |
| Lingala | Jill’s daughter | F | Juvenile | 2003 |
| Zuani | Liboso’s mother; Nayembi’s grandmother | F | Adult | ~1990 |
| Liboso* | Zuani’s daughter; Nayembi’s mother; Makasi’s sister | F | Adult | 1997 |
| Nayembi | Liboso’s daughter; Zuani’s granddaughter; Makasi’s niece | F | Infant | 2006 |
| Hortense* | Zamba’s mother; Hongo’s mother | F | Adult | ~1978 |
| Zamba | Hortense’s son; Hongo’s brother | M | Adult | 1998 |
| Hongo | Hortense’s son; Zamba’s brother | M | Infant | 2006 |
| Mobikisi | - | M | Adult | ~1980 |
| Kumbuka | - | F | Adult | 1999 |

**a)**

| Name | Kinship | Sex | Age class | Year of birth |
| --- | --- | --- | --- | --- |
| Daniela* | Diwani and David’s mother | F | Adult | 1968 |
| Ukela | Nakala’s mother | F | Adult | 1985 |
| Khaya* | - | F | Adult | 2001 |
| Lingala* | - | F | Adult | 2003 |
| Nakala | Ukela’s daughter | F | Infant | 2007 |
| Kirembo |  | M | Adult | 1992 |
| Diwani | Daniela’s son | M | Adult | 1996 |
| David | Daniela’s son | M | Adult | 2001 |
| Kelele | - | M | Adult | 2004 |
| Lucy* | - | F | Adult | 2003 |

**b)**

**Supplementary Table S2.** List of body postures, gestures and facial expressions involved in sexual invitations (for the details of some gestures see also Pollick and de Waal, 2007 and Douglas and Moscovice 2015)

| **Genito-Genital Rubbing** | between two females in ventro-ventral, dorso-dorsal or ventro-dorsal position. Females rub their genital each other with lateral movements (only *Pan paniscus*) |
| --- | --- |
| **Sexual Invitation** | different in male and female. Male sits and slaps the feet on the ground. The legs are opened. He oscillates and shows its penis (identical in *Pan paniscus* e *Pan troglodytes)*  Female walks in front of male while staring it, then she stops and restarts. In *Pan paniscus,* the female can lying down or crouching in front of the male |
| **Sexual Crouching** | crouching position used during sexual contacts |
| **Sexual Presenting** | an individual gets closer to another one showing its genital area from behind |
| **Bent wrist** | Flexing the wrist while holding the back or side of hand out toward another individual; contact possible (from Pollick and de Waal, 2007) |
| **Reach out down** | Holding out a hand toward another individual by extending the arm, wrist, and hand in more or less horizontal position, and stretching the fingers while palm is facing downwards; other individual is not touched |
| **Reach out side** | Same as reach out down except the palm of the hand is directed sideways |
| **Reach out up** | Same as reach out down except that the open palm of the hand is directed upward |
| **Shake wrist** | Shaking the hand vigorously with flexible wrist towards another subject. It can be repetitive |
| **Pout face** | Lips are pursed and protruded but remain in contact near the mouth corners and are parted in middle (duck face) |

**References**

Douglas, P. H. & Moscovice, L. R. 2015. Pointing and pantomime in wild apes? Female bonobos use referential and iconic gestures to request genito-genital rubbing. Scientific Reports, 5, 13999.

Pollick, A. S., & De Waal, F. B. (2007). Ape gestures and language evolution. Proceedings of the National Academy of Sciences, 104(19), 8184-8189.

**
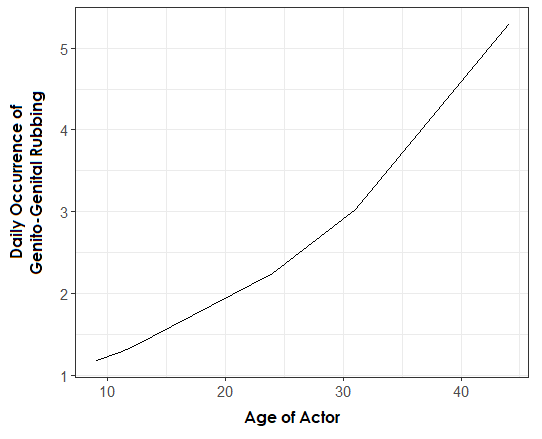
Supplementary Figure S1** Effect of actor’s age on the daily occurrence of Genito-Genital rubbing.

**
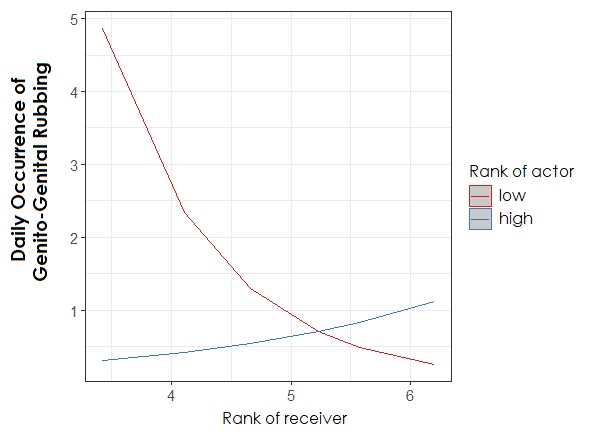
Supplementary Figure S2.** Marginal effects of the two-way interaction of *rank_act_*rank_rec_* on the daily occurrence of Genito-Genital rubbing. *Rank_act_* is plotted at its minimum (Normalized David’s Score 3.42) and maximum values (Normalized David’s Score 6.2)
